# Enhanced Materials from Nature: Nanocellulose from Citrus Waste

**DOI:** 10.3390/molecules20045908

**Published:** 2015-04-03

**Authors:** Mayra Mariño, Lucimara Lopes da Silva, Nelson Durán, Ljubica Tasic

**Affiliations:** 1 Chemical Biology Laboratory, Institute of Chemistry, Organic Chemistry Department, State University of Campinas, P.O. Box 6154, Campinas 13083-970, Brazil; E-Mails: mayra.bohorquez@iqm.unicamp.br (M.M.); lucimaraeq@gmail.com (L.L.S.); 2 Chemical Biology Laboratory, Institute of Chemistry, Physical-Chemistry Department, State University of Campinas, P.O. Box 6154, Campinas 13083-970, Brazil; E-Mail: duran@iqm.unicamp.br

**Keywords:** nanocellulose, citrus waste, enzymatic hydrolysis, *Xanthomonas axonopodis* pv. *citri*, cellulose crystallinity index, nuclear magnetic resonance

## Abstract

Nanocellulose is a relatively inexpensive, highly versatile bio-based renewable material with advantageous properties, including biodegradability and nontoxicity. Numerous potential applications of nanocellulose, such as its use for the preparation of high-performance composites, have attracted much attention from industry. Owing to the low energy consumption and the addition of significant value, nanocellulose extraction from agricultural waste is one of the best alternatives for waste treatment. Different techniques for the isolation and purification of nanocellulose have been reported, and combining these techniques influences the morphology of the resultant fibers. Herein, some of the extraction routes for obtaining nanocellulose from citrus waste are addressed. The morphology of nanocellulose was determined by Scanning Electron Microscopy (SEM) and Field Emission Scanning Electron Microscopy (FESEM), while cellulose crystallinity indexes (CI) from lyophilized samples were determined using solid-state Nuclear Magnetic Resonance (NMR) and X-Ray Diffraction (XRD) measurements. The resultant nanofibers had 55% crystallinity, an average diameter of 10 nm and a length of 458 nm.

## 1. Introduction

Lignocellulose, which forms part of plant cell walls, is the most common carbon deposit produced by Nature and the principal component of organic waste. This biomass is a non-edible residue from the agricultural industry that is gaining importance as a source for the energy and biomaterials sectors, despite previously being considered an environmental problem. Either for biofuel production or for obtaining cellulose nanofibers, the breakdown of the cross-linked elements in the raw material (lignin, cellulose, pectin and hemicellulose) has to occur to increase accessibility to the cellulose microfibrils [1]. Lignocellulose presents semicrystalline parallel aggregates of cellulose that form microfibrils with a width to length ratio of 1:10 to 1:20 µm [2]. An additional step coupled to the purification process is necessary to dislocate the fibers cluster, and give after this step a nanomaterial with enhanced mechanical and thermal properties. The cellulose nanofiber (CNF) resulting from processing this biomass presents a larger crystalline region and a higher specific surface area (approximately 150 m^2^/g), corresponding to nanofibrils with diameters less than 100 nm and an average length of 1 µm, also called microfibrillated cellulose nanofibers (MFC). Therefore, the isolation and nanofibrillation processing of the crystalline region of cellulose results in a lightweight porous material with remarkable mechanical resistance that has an axial elastic modulus of 130–150 GPa and a tensile strength of 0.8–10.0 GPa. Furthermore, this bionanomaterial enhances the barrier properties of nanocomposites, and these properties are especially important for the development of high performance nanocomposites [3], which have potential applications that include reinforcement of the biodegradable composite films used for food packing and paper printing [4,5]. The development of several microscopy and spectroscopy techniques allowed us to collect unambiguous information on the nanostructure of lignocellulose and to analyze the effects of the different stages used during the isolation and nanofibrillation of cellulose. Typically, physicochemical pretreatment breaks down the supramolecular cell wall structure, thus increasing accessibility to the polysaccharide components of the raw lignocellulose [1,6]. The addition of aqueous sodium hydroxide, a bleaching agent, and subsequent hydrothermal compression, also called the steam explosion method, is the most efficient pretreatment for plant fibers based on partial lignin and hemicellulose extraction. By inducing an expanded state, the interfibrillar space increases, and the alkaline medium breaks the hydrogen bonds between fibers (mercerization), increasing the number of amorphous domains. This chemical treatment also increases the specific superficial area and the absorption capacity of cellulose and partially changes crystalline cellulose domain I to crystalline cellulose domain II, a cellulose form with antiparallel chains of cellulose homopolymer [7]. For biomass that is highly recalcitrant to hydrolysis, such as wood or sugarcane bagasse, heat-compressed water treatment has achieved more efficient biomass treatment results when used at 180–200 °C [8].

The resultant mercerized fiber exhibits an increase in semicrystalline cellulose content and a high susceptibility to enzymatic hydrolysis once the cellulose reducing ends with a lower degree of crystallinity are exposed. After reducing end exposure, hydrolysis or saccharification can occur, which involves breakdown of the β-(1,4)-glycosidic bonds that link the d-glucopyranoside units in plant cellulose fibrils (approximately 1–10 nm thick). Typically, this depolymerization process can be achieved with acid solvents or by enzyme addition. Both of these routes enrich the crystallinity of cellulose, although acid hydrolysis, which is more selective at solubilizing amorphous cellulose, shows better results for increasing the crystallinity of the remaining cellulose than does enzymatic hydrolysis. However, acid hydrolysis yields rod-like crystallites, called cellulose nanowhiskers (CNW) or cellulose nanocrystals (CNC), with a shorter average length and lower tensile strength. This process also involves additional environmental impact of the effluents and the degradation of matrix components under harsh conditions [9,10,11,12]. The most common hydrolyzing agent is sulfuric acid, which stabilizes the nanowhisker suspension by electrostatic repulsion avoiding fibril aggregation, but modifies the native cellulose surface [3]. Moreover, acid hydrolysis under milder conditions using hydrochloric and oxalic acids has been successfully used for pineapple, banana and jute lignocellulose materials [13]. The nanostructuration or dispersion of microcrystalline cellulose (CMC) is typically the step that requires additional energy consumption, because it is generally achieved by various mechanical treatments, such as cryocrushing, high intensity waves (ultrasonication) and high-pressure homogenization [14,15]. To reduce energy consumption, an alternative has been proposed: an enzymatic hydrolysis step that provides a solid remnant with a high content of microcrystalline cellulose, with the resultant liquor content used for the production of second generation ethanol [16,17].

Secondly, the economic impacts of nanoscale cellulose fibrils involve producing the raw material source. Diverse chemical/biochemical and mechanical treatments have been utilized to obtain nanocellulose, and its morphology depends on both the biomass processing and the fiber source. Many agricultural byproducts are being used to isolate cellulose fibrils. Thus far, the motivation to explore more locally obtained residues has increased interest in nanocellulose production using cereal byproducts such as wheat straw [18,19], soy hulls [18], soybean straw [20], sorghum fibers [21] and rice straw [22,23], as well as other crop residues such as cassava bagasse [24], banana fibers [13,25,26,27,28], pineapple leaves [13,29], sugarcane bagasse [30,31,32], cornstalks [33], cornhusks [34], oil palm biomass [35], grape hulls [36], and orange bagasse [37]. In fact, citrus waste is an interesting agricultural waste that has already been processed to obtain nanofibers of microcrystalline cellulose with enhanced properties compared to cellulose. Currently, Brazil is the world’s principal producer of orange juice, generating 20–22 million tons of oranges annually, and only half of these fruits are used during juice manufacturing. The citrus waste byproduct contains approximately 15.2% cellulose, 18.2% hemicellulose and 24.6% pectin in the dry biomass and is commonly exploited as a food supplement in pellets for cattle, for example. The remaining content (41.6%) is represented by soluble sugars (15%–20%), proteins (6.5%–8.0%), starch (3.8%), ashes (3.5%) and minor components (lignin, fats, flavonoids, *etc*.) [38,39]. In this study, we present the characterization of NFC from citrus-waste biomass (CB) subjected to enzymes from *Xanthomonas axonopodis* pv. *citri* strain 306 (IBSBF 1594), a bacterial species isolated from infected citrus fruits that can be fatal to citrus trees. These phytopathogenic bacteria were detected in Sao Paulo state in the late 1990s after causing citrus canker disease, but their specific digestion of orange fibers has been proposed as a strategy to obtain CMC from citrus-waste biomass, a significant renewable source of nanocellulose [17,39]. In this study, we explored the structural and morphological characteristics of cellulosic material obtained by hydrolysis of CB in mild conditions. The morphological changes and the purity the cellulose fibrils after each stage of non-cellulosic component removal from this biomass were addressed by SEM and FESEM, while the structural analysis was carried out by solid state NMR spectroscopy, XRD and FTIR.

## 2. Results and Discussion

### 2.1. Effect of Enzymatic Hydrolysis on the Morphology of Cellulose Fibers

#### 2.1.1. Morphological Changes

**Figure 1:**
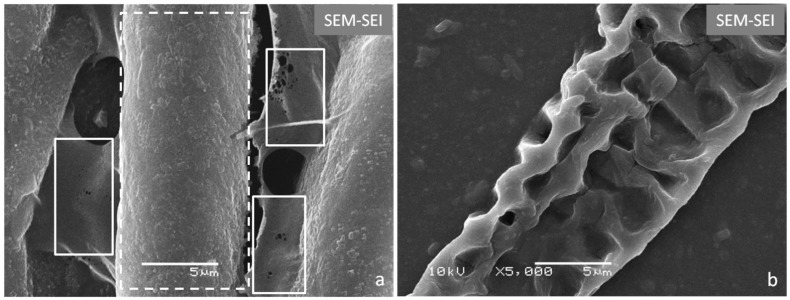
Scanning electron micrographs of the Citrus-waste Biomass (CB) before and after enzymatic treatment: (**a**) CB sample pretreated with NaOH (4% w/v) at 120 °C for 20 min and NaClO_2_ (1.7% w/v) for 30 min at 120 °C and pH 4.5; (**b**) CB sample pretreated as in (a) and after *Xanthomonas axonopodis* pv. *citri* enzymatic action for 48 h at 45 °C at pH 5.0.

The initial physicochemical step of swelling after adding a sodium hydroxide solution is associated with fractionation of the pectin-lignin-hemicellulose matrix and involves disruption of the cellulose bundles or macrofibrils in the supramolecular assembly of the biomass. Hydrothermal treatment increases enzymatic accessibility to the reducing ends of cellulose by generating space in the cellulose bundles [7]. Secondary Electron Image (SEI) micrographs show that after chemical pretreatment by delignification and subsequent bleaching, citrus waste biomass fibers maintain a relatively closely packed structure, while their surfaces are covered with residual material (Figure 1a). The effect of enzymatic digestion was verified by the complete degradation of the thinner and most vulnerable cellulosic material (boxed areas in Figure 1a) and the emergence of several defects/holes on the sturdier fiber surface (Figure 1b and dashed area in Figure 1a). In accordance with previous studies from our research group [39], *Xanthomonas axonopodis* pv. *citri* (*Xac* 306) enzymes are very active on citrus waste biomass. These bacteria, which cause citrus canker disease, produce an enzyme cocktail that contributes to biomass degradation because of the hydrolytic activities of the different enzymes.

Henriksson *et al*. [40] investigated the effects of enzymes on biomass degradation. An auxiliary swelling of fibers that facilitated their disintegration has been observed. Their work has also shown that energy consumption of mechanical treatments can be reduced by the action of specific enzymes, once the disintegration of entangled networks present in cellulose fibers can be assisted by hydrolases [41]. The MFC with high degree of polymerization are the result of hydrolysis in mild conditions coupled to high shear forces and have been highlighted like a reinforcement agent with interesting properties [12,13,14,15,28,32,41].

**Figure 2:**
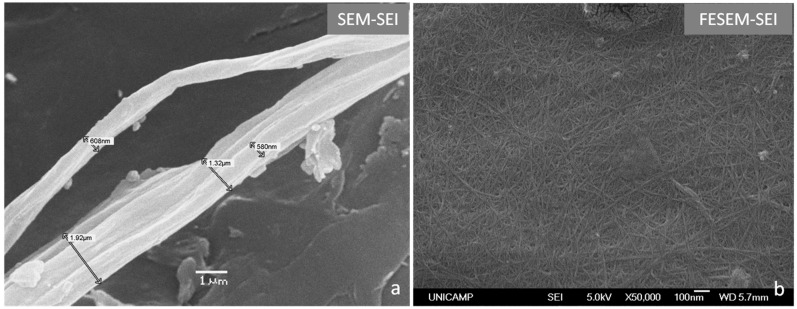
Scanning electron micrographs showing the hydrolytic effects of *Xanthomonas axonopodis* pv. *citri* enzymes on CB: (**a**) fibers and bundle of fibers; (**b**) nanocellulose fibers in detail.

In this study, the enzymatic treatment also had a marked effect, especially on fiber bundles that became detached from the other bundles (Figure 2a), resulting in additional unstructured, independent fibers. These fibers with diameters of approximately 600 nm are present in some areas of the sample holder. However, analysis by Field Emission Scanning Electron Microscopy (FESEM) enabled observation of cellulose nanofibers throughout the sample holder (Figure 2b), indicating that nanocellulose is the most abundant fiber obtained and that enzymatic hydrolysis using this bacterial enzyme cocktail was very efficient. This is evidence for how enzymatic hydrolysis can improve cellulose nanofibrillation, because prior to hydrolysis, only micrometer-length fibers (Figure 1a) were observed. Length and width in Figure 2b were determined through measurements on visible parts of nanofibers (see Supplementary Information, Figure S1) because the total length cannot be determined since the cellulose nanofibers are matted and not individualized as seen in FESEM image. However, it is possible that length, and thus aspect ratio of nanocellulose are greater than the reported.

More accurate size determination is an important question to be answered by future work, considering reducing the NCF concentration dropped onto the sample holder. Cellulosic nanofibers have a rod-like structure with an average length of 458 ± 115 nm and a width of 10 ± 3 nm, generating and average aspect ratio of 47 ± 18 nm, which is considered high. However, it is possible that length, and thus aspect ratio of nanocellulose are greater than the reported. Generally, microbial and/or enzymatic treatments of cotton and sugarcane fibers produce nanocellulose with aspect ratios of 43 ± 13 and 55 ± 21 nm, respectively. The cotton nanofibers exhibit average lengths of 120 ± 36 nm, and sugarcane bagasse nanofibers have lengths of 255 ± 83 nm. Therefore, the sizes of nanocellulose obtained from CB are consistent with other reported data, confirming that CNF can be obtained from several renewable sources [12,32]. These data also indicate that CB nanofibers can be efficiently used as reinforcing agents in composite materials, because the long fibers typically provide higher gains in mechanical properties.

#### 2.1.2. Structural Changes in Cellulose Fibers during the Multi-Stage Procedure

**Figure 3:**
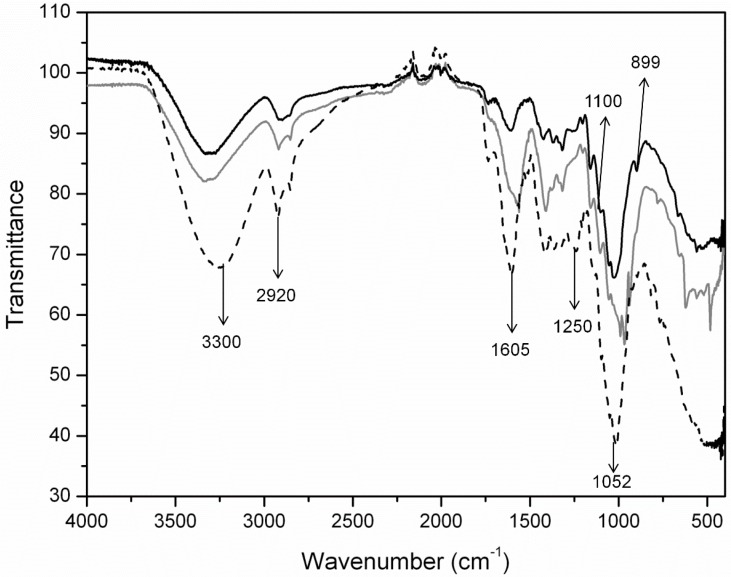
FTIR spectra of: the raw Citrus-waste Biomass (CB) (dashed line), nanocellulose obtained from CB after two-stage procedure of delignification and bleaching (gray), and after an additional enzymatic hydrolysis step (black).

In addition to the dimensions of fibrils in cellulose aggregates, crystallinity is a key parameter that determines the morphology of the cellulose biopolymer. Crystallinity Index (CI) values greater than 60% have been reported for most nanocellulose materials [8]. The CI results in this study are correlated with the changes in the substrate owing to enzymatic digestion by the cellulases present in the *Xac* enzyme cocktail. To analyze the cellulosic material extraction, the structural characteristics of the raw material, fibers after chemical treatment and fibers after chemo-enzymatic treatment were ascertained by Fourier transform infrared spectroscopy (FTIR). Figure 3 shows the FTIR spectra of the raw material and material treated by NaOH delignification and bleaching at 120 °C. The third spectrum shows the material resulting from enzymatic hydrolysis of CB by the *Xac* enzymes, as well as the aforementioned chemical treatment.

The cellulose I profile is common to both products and is denoted by intramolecular hydrogen bonding between the hydroxyl groups on C-2 and C-6 of the pyranose rings (3330 cm^−1^), the CH_2_ symmetric bending at C-6 (1410–1424 cm^−1^), the CH_2_ wagging bending at C-6 (1318 cm^−1^), the COH in-plane bending at C-6 (1203–1205 cm^−1^) and the C-O-C motions of the β-glycosidic linkages (1102–1105 cm^−1^ and 1054–1056 cm^−1^) [42]. Additional bands in the carbonyl-stretching region, which account for 1734 and 1605 cm^−1^, are assigned to the ester and acetyl groups in hemicellulose and lignin [28,43,44]. As shown in Figure 3a characteristic band for carboxylate compounds is representative for all the materials and indicates only a partial removal of hemicellulose, lignin and pectin by both extraction routes. In this case, a CI determination by FTIR, as reported by Nelson *et al*. [43], is not recommended due to the co-existence of amorphous compounds in addition to hemicellulose.

However, the intensity decrease of the band at 1250 cm^−1^ (C-O stretching vibration of hemicellulose) is an indication of the effectiveness in the removal of hemicellulose. When compared the bands at 1250 cm^−1^ for the treated materials and the band in the FTIR spectrum of the CB raw material, a remarkable removal of hemicellulose can be observed. The cellulose contents are addressed in Table 1 as extraction yields obtained in each step of CB treatment.

Another significant structural variation noted is related to the stretching involving C-O-C and C-C-H at C-5 and C-6 that is observed at 893–902 cm^−1^. While the enzyme-treated material presents the characteristic band for β-glycosidic linkages between glucose units at 899 cm^−1^, the chemical-treated material lacks this band and shows three additional vibrations at higher wave numbers (990, 968 and 940 cm^−1^). Bands in this frequency region are attributed to C-O stretching of polysaccharides owing to residual hemicelluloses, which can’t be digested by the enzymatic cocktail of *Xac* enzymes [28,44]. The remaining content of hemicellulose and/or pectin corresponded to about 16% of the final nanomaterial (Table 1). Additionally, the band around 1375 cm^−1^ is attributed to C-H bending in cellulose with the high crystallinity index, whereas the band around 2900 cm^−1^ is characteristic for aliphatic C-H stretching of hemicellulose and cellulose [43,44].

**Table 1:** Percentage of cellulose and crystallinity indexes (CI) calculated for the cellulosic materials using XRD and NMR data.

Sample	Cellulose Content (%)	CI by XRD	CI by NMR
Raw material (CB)	16.46 ± 0.84	0.16	-
NaOH-treated fibers	52.50 ± 1.35	-	-
NaOH-treated and bleached fibers	76.75 ± 0.51	0.50	0.36
Chemo-enzymatic treated fibers	83.80 ± 0.48	0.63	0.55

**Figure 4:**
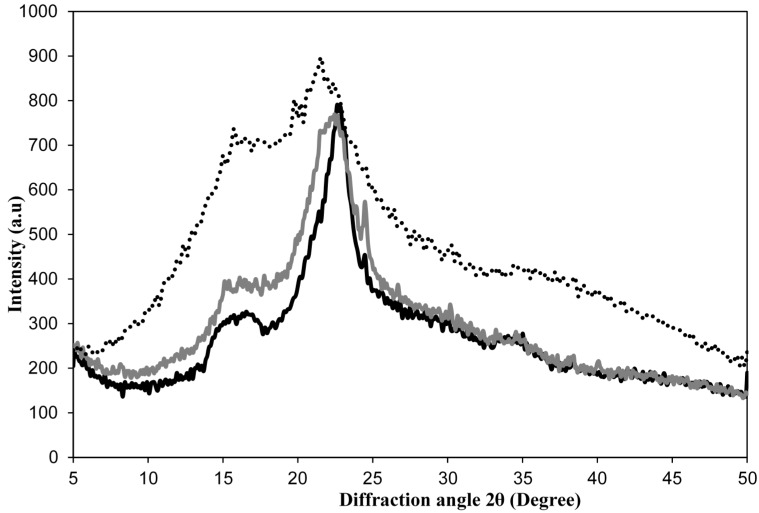
X-ray diffractograms of Citrus-waste Biomass (CB) as raw material (dashed line), nanocellulose obtained by the physicochemical method of delignification and bleaching using NaOH and NaClO_2_ (gray) and after enzymatic treatment for 48 h at 45 °C (black).

X-ray diffractograms provided data for the CI changes in the cellulose materials after physicochemical and biological treatments. Regarding the X-ray diffraction patterns, the cellulose lattice presents at least four crystalline peaks involving an amorphous region. The curve corresponds to the 16.4° peak (101 plane), which can be separated into amorphous and crystalline portions by deconvolution, whereas the highest peak at 22.4° (002 plane) is typically used in the calculation of the CI [45]. As shown in Figure 4, peaks that correspond to the typical cellulose I structure were observed in the raw material, Citrus-waste Biomass (CB), and in the treated materials. And the peak intensities of the crystalline cellulose peak (22.4°) increased with the increased number of process steps. Herein, we used the peak height method of Segal *et al.* [46] a simple calculation of the ratio between the 002-peak intensity (*I_002_*) and the minimum intensity (plateau) between the 002 and 101 peaks (I_AM_). This empirical method for determining the crystallinity of cellulose is useful solely as a means of comparison, owing to the inherent approximation of the amorphous portion. A more accurate estimation of the height of the amorphous peak is achieved by software that applies the deconvolution method. Lorentzian or Gaussian functions are common assumptions for the shapes of peaks in curve-fitting, resulting in greater heights of amorphous peaks, which leads to a higher contribution of amorphous cellulose and thus lower CI values [47]. However, in this case, comparison of the CI values from the diffraction profiles exhibited an increase of approximately 18% in the crystallinity of the enzyme-treated material (Table 1). As shown by Figure 4, a marked decrease in the intensity of the amorphous region between 16.4° and 18.0° can be attributed to enzymatic hydrolysis, while the crystalline 002 region showed negligible variation in signal intensity but a decrease in peak area. These results are associated with cellulose and hemicellulose/pectin hydrolysis, as indicated by FTIR spectra and electronic micrographs. The remaining hemicellulose and/or pectin contents are correlated with the amorphous areas.

Other reports [12,32] present data on cotton nanofibers, which have an average crystallinity index of 78.4%, and sugarcane bagasse nanofibers (crystallinity index of 74.0%), which both have higher CIs than these obtained for CB nanocellulose.

**Figure 5:**
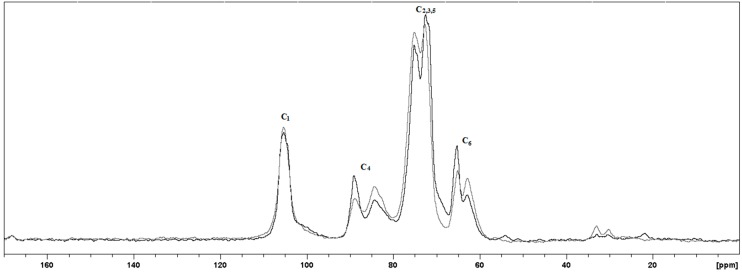
CP/MAS NMR spectra of nanocellulosic fibers obtained from Citrus-waste Biomass (CB) that was enzymatically treated for 48 h at 45 °C (black) and from chemically treated CB (delignification and bleaching at 120 °C; gray).

The CP/MAS ^13^C-NMR spectra (Figure 5) revealed the characteristic peaks of cellulosic material with marked variations in the C-4 and C-6 areas. The variations in the C-4 peaks are specifically employed to evaluate the CI of cellulose I. The C-4 peak at 89 ppm is attributed to the ordered structure, whereas the C-4 peak at 84 ppm is assigned to a less-ordered fibril surface. For the CI calculation, line-fitting by the deconvolution method is needed, followed by integration of the total C-4 area corresponding to 80–93 ppm. However, the region in the downfield side (87–93 ppm) is the principal contributor to the crystalline phase of the cellulose I structure. For the resonances from the other carbons in the pyranose ring in Figure 5, C-2, C-3 and C-5 overlap in the 72–76 ppm region, C-6 is assigned to the 62–65 ppm region in a split similar to C-4, and C-1 is at 105.4 ppm with a singlet signal corresponding to allomorph cellulose I_α_ [47].

The ratio of the C-4 crystalline area at 87–93 ppm and the total line-fit area of each spectrum shown in Figure 5 led to the following CI values: 0.55 for the enzymatically treated biomass, 0.36 for the chemically treated bagasse and 0.29 for the NaOH-treated citrus waste biomass. As discussed previously, the lower crystallinity values, compared to the CI values obtained by XRD, are due to the rough approximation of the amorphous phase intensity, which is assumed when using the peak height method.

As a result of the treatments, the CB residue was enriched in crystalline cellulose compared to the starting material. The efficiency of bleaching substantially increases the crystallinity by 7.0%, while enzymatic treatment achieved an increase in crystallinity of approximately 13%. The delignification and bleaching processes used were suitable for increasing the citrus waste biomass digestibility through partial hydrolysis of hemicellulose and pectin, while the removal of lignin was achieved using sodium hydroxide [44]. The extent of amorphous cellulose solubilization by microbial or enzymatic hydrolysis has been controversial due to the selectivity of cellulases. Satyamurphy *et al*. [12] isolated cellulose nanofibers from cotton by using fungal hydrolysis, and these nanofibers had a lower crystallinity than those isolated by using acid hydrolysis. Park *et al.* [45] attempted to understand cellulose performance in terms of CI changes as a result of the preferential solubilization of amorphous cellulose by cellulases. According to the authors, a slight CI increase of 2%–3% after enzymatic or bacterial hydrolysis of cellulose suggests that high hydrolysis rates by cellulases are occurring in the highly amorphous biomass. The NMR spectrum of the chemically treated nanocellulose (Figure 5) shows resonance peaks between 20 and 35 ppm from the presence of the methyl OCOCH_3_ carbons in pectin. Additionally, the 1730 cm^−1^ band in the FTIR spectrum (Figure 3) is correlated with residual acetylated pectin or hemicellulose. However, the observed CI increase occurred due to the action of the *Xac* enzymes on the loose cellulose ends and pectin [48]. The *Xac* enzymes include cellulases and pectinases that act on less-ordered regions, which means that there is better substrate accessibility in these regions [39].

Because of the increase in the amorphous domain and easier access to the cellulose reducing ends, the reactivity of amorphous cellulose is slightly higher than that of crystalline cellulose; therefore, saccharification is enhanced at a lower crystallinity index (CI). Thus, enzymatic hydrolysis increases the CI until the endoglucanase is saturated: endoglucanase is the cellulase that starts the synergistic process of hydrolysis by binding and cleaving the reducing ends of amorphous cellulose chains, while exoglucanase, also called cellobiohydrolase, acts sequentially by binding the ends of crystalline lattices. The lower hydrolysis rates of the crystalline regions may be associated with the crystalline domains being a more resistant to fractionation than the amorphous regions; thus, the generation of entry points for exoglucanase by dislocation of the layered cellulose crystals may occur less often because of crystalline domains larger cohesion energy density [49].

## 3. Experimental Section

### 3.1. Pretreatment of Citrus-Waste Biomass Fibers

Bagasse from the *Citrus sinensis* (L) *osbeck* variety was ground, sieved to a particle size of 0.85–1.15 mm and oven-dried at 120 °C. The resultant biomass was pretreated by delignification using NaOH (4% w/v, 1:25 ratio) and subsequent bleaching with 1.7% w/v sodium chlorite at pH 4.5 as described by Tsukamoto *et al.* [17]. For each step, the samples were maintained at 120 °C under a pressure of 1 atm for 30 min, and the residual reagents were removed by vacuum filtration with hot water.

### 3.2. Enzymes from Xanthomonas axonopodis pv. citri (Xac)

The production of the bacterial enzyme cocktail was performed using *Xanthomonas axonopodis* pv. *citri* strain 306 (IBSBF 1594), according to the incubation and protein extraction procedures described by Awan *et al.* [38]. A 24 h inoculum of the bacteria was prepared in Luria-Bertani medium with sodium carboxymethylcellulose (5 g·L^−1^) and glucose in a 1:2 ratio as the carbon sources and grown at 32 °C and pH 7.0 under shaking incubation (90 rpm). After centrifugation at 30,678*× g* for 30 min, the cellular mass was suspended in sterile extraction buffer (10 mmol·L^−1^ Tris–HCl, pH 8.0, 10 mmol·L^−1^ NaCl, 50 mmol·L^−1^ EDTA) at a 1:2 w/v ratio. The suspension remained at −80 °C for 1 h before a 15 min lysis by sonication in an ice bath. Then, the lysate was centrifuged at 30,678*× g* for 15 min at 4 °C, and the supernatant was dialyzed against water using a cellulose membrane (3.5 kDa cut-off, Fisher Scientific, Pittsburgh, PA, USA) until a constant mass was achieved. The solution obtained by this procedure had a protein concentration of approximately 1.0 ± 0.2 mg mL^−1^, which was determined using a Bio-Rad assay [50].

### 3.3. Enzymatic Hydrolysis and Isolation of Cellulose Nanofibers

The fibers resulting from the pretreatment process were diluted in water until 17% w/v was achieved, and this solution was adjusted to pH 5.0 for enzymatic hydrolysis. Then, 5 mg of the *Xac* protein solution were added per gram of CB. Enzymatic hydrolysis was performed at 45 °C and 90 rpm for 48 h. The hydrolyzates were then filtered by vacuum filtration before dilution of the bioresidue to approximately 1% w/v, and the dilute filtrate was subsequently dialyzed with water in a cellulose membrane (6–8 kDa cut-off, Fisher *Sci*). The sonication of the fibrous material was performed with a tip that delivered a power of 75 W (VCX-750, Vibra-Cell, Newtown, CT, USA) for 12 min. This sonication process was performed in triplicate.

### 3.4. Microstructure Analysis

The morphological characteristics of the citrus-waste biomass (CB) cellulose fibers and nanofibers, resulting from chemical and enzymatic hydrolyses, were investigated using JEOL JSM-6360 LV SEM and JEOL JSM-6340LV FESEM microscopes (JEOL, Akishima, Tokyo, Japan). The sonicated cellulose dispersion was dropped onto a sample holder, dried at room temperature and coated with gold or platinum using an MED 020 Sputter (BalTech, Balzers, Liechtenstein). Images were obtained using a 5 or 10 kV accelerating voltage and a secondary electron detector. The mean fiber diameters and lengths were determined from the FESEM image using ImageJ 1.49o, National Institutes of Health, Bethesda, MD, USA, 2015). For this purpose, 20 segments were randomly selected.

### 3.5. FTIR Spectroscopy Analysis

The structural changes in CB, as determined by FTIR spectroscopy, were investigated in freeze-dried samples in a CARY 630 spectrophotometer (Agilent Technologies, Santa Clara, CA, USA). Each spectrum was obtained by accumulating 128 scans at a resolution of 4 cm^−1^ in the 4000 cm^−1^ to 400 cm^−1^ range.

### 3.6. NMR Spectroscopy Analysis

Lyophilized nanocellulose and cellulose samples obtained from citrus-waste biomass (CB) were used for all of the structural analyses by solid state NMR. The ^13^C-NMR spectra were collected on a Bruker AMX-300 MHz instrument (Bruker, Billerica, MA, USA) operating at 7.05 T and 75.47 MHz with cross-polarization and magic angle spinning (CP/MAS). Acquisition of the CP pulse sequence was performed using a 3000 Hz MAS rate, a 90° pulse for 1.5 ms and a 800-ms contact pulse. The number of scans was 10,000 and 3.0 s delay was used for repetitions.

The Crystallinity Index (CI) was determined by line fitting using the deconvolution method assuming a Lorentzian line shape for the cellulose C-4 peaks. The C-4 region corresponding to 86–92 ppm was assigned to crystalline cellulose, while the cellulose C-4 total area (79–92 ppm) was used in the procedure for calculating the CI, as reported by VanderHart and Atalla [47].

### 3.7. XRD Analyses

Lyophilized nanocellulose and cellulose samples obtained from citrus waste were analyzed using XRD. A Shimadzu XRD-7000 X-ray diffractometer (Shimadzu, Columbia, MD, USA) operating at 40 kV and 30 mA was used to obtain the diffraction profile at 2° per min, and data were recorded using a copper (Kα) radiation source and a secondary monochromator. Diffraction patterns were scanned over a 2θ range of 5.0°–50.0°, and the CI were calculated using the following equation, according to Segal *et al*. [46]:

CI = (*I_002_* − *I_AM_*)/*I_002_* (1)

where *I_002_* is the height of the 002 peak (at 2θ of approximately 22.6°) and *I_AM_* is the minimum height (plateau) between the 002 and 101 peaks (at 2θ of approximately 18°).

**Figure 6:**
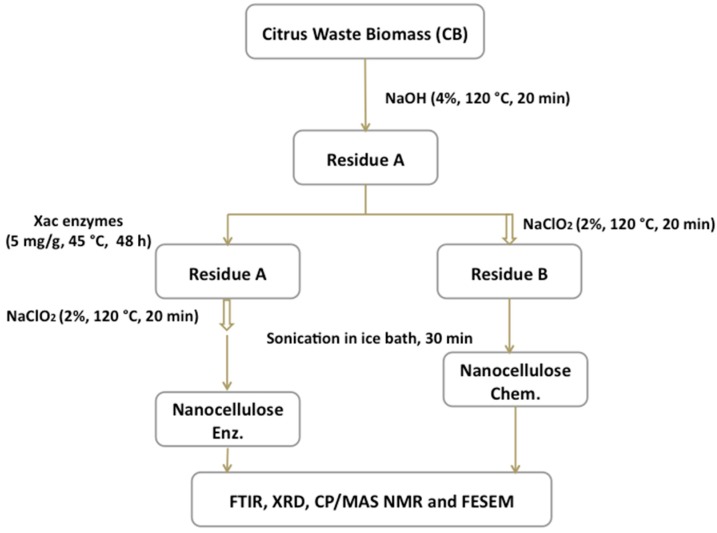
Flowchart illustrating the key steps in the Citrus-waste Biomass (CB) processing used to produce nanocellulose fibers.

In Figure 6, we present a flowchart illustrating the key steps for obtaining citrus waste biomass nanocellulose, such as the swelling and bleaching of chemically obtained NFC (Nanocellulose Chem.) and the swelling, enzymatic treatment and bleaching of biochemically obtained NFC (Nanocellulose Enz.).

### 3.8. Cellulose Content

The yield of each step used for cellulose extraction was estimated according to the following protocol of ANKOM technology: acid detergent fiber in feeds − filter bag technique (for A200 and A200I):

Cellulose percentage = [weight of dried bag with fiber after extraction − (W_1_ × C_1_)]/W_2_ × 100 (2)

where W_1_ is the bag tare weight, C_1_ is the blank bag correction (ratio between average of final oven-dried weight and weight of the original blank bag) and W_2_ is the sample weight. The measurements were conducted in three replicates.

## 4. Conclusions

The morphological changes in citrus waste biomass fibers were promoted by a three-step physicochemical and enzymatic procedure. Utilization of a low NaOH concentration enabled preservation of the cellulose I structure and aided in the hydrolysis of the amorphous components with cost-efficient enzymes from *Xanthomonas axonopodis* pv. *citri* (*Xac*). Subsequent sonication allowed us to obtain nanocellulose fibers with a high aspect ratio, thus adding great value to a substance previously regarded as agricultural waste. Spectroscopic and X-ray analyses provided evidence of an increase in ordered cellulose content, which was achieved by solubilization of the amorphous cellulose, hemicellulose and/or pectin present in this bioresidue and the contribution of the enzymatic cocktail to digestion of this biomass. An enrichment of 7% in cellulose nanofibers content and the CI increase of 13% have been achieved by enzymatic hydrolysis. After sonication, cellulose nanofibers with approximately 55% crystallinity and an average diameter of 10 nm were observed. Therefore, citrus waste biomass possesses high potential as a renewable substance for fabricating important nanobiomaterials such as nanocellulose.

## Supplementary Materials

Supplementary materials can be accessed at: http://www.mdpi.com/1420-3049/20/04/5908/s1.
