# Supplementary material for: Enhanced Materials from Nature: Nanocellulose from Citrus Waste
Source: Molecules. 2015 Apr 3;20(4):5908–23. doi: 10.3390/molecules20045908 (PMC6272572; doi:10.3390/molecules20045908)
Supplement: Supplementary file 1 [file molecules-20-05908-s001.pdf]

Supplementary Materials

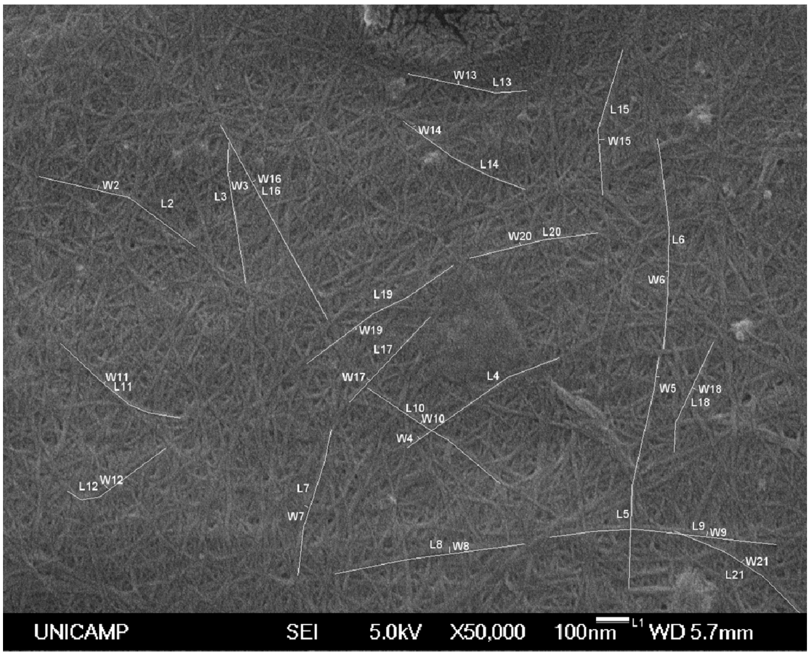

Figure S1. Field emission scanning electron micrographs with 20 segments randomly selected for length (L) and width (W) nanofibers determination using ImageJ 1.49o software

Table S1. Width (W) and length (L) measurements of 20 segments aforementioned and the calculation of their aspect ratio. The length highlighted in orange is performed on the calibration from the bar scale of the micrograph.

Segment	Length (nm)	Width (nm)	Aspect Ratio
1	100.000		
2	533.769	11.820	45.16
3	433.422	5.805	74.66
4	541.493	11.556	46.86
5	710.628	9.615	73.91
6	641.515	8.333	76.98
7	457.462	9.465	48.33
8	584.243	17.994	32.47
9	696.349	13.598	51.21
10	498.519	6.346	78.56
11	442.774	8.209	53.94
12	357.139	15.129	23.61
13	369.831	9.615	38.46
14	429.830	6.047	71.08
15	454.102	15.398	29.49
16	676.541	8.905	75.97
17	357.760	8.719	41.03
18	360.400	11.029	32.68
19	538.190	8.333	64.59
20	400.408	10.746	37.26
21	459.058	10.435	43.99
Average	458.260	9.615	47.60
Standard Deviation	114.842	3.212	18.01
